# Alternative meta-analysis of behavioral interventions to promote action on climate change yields different conclusions

**DOI:** 10.1038/s41467-020-17613-7

**Published:** 2020-08-05

**Authors:** Sander van der Linden, Matthew H. Goldberg

**Affiliations:** 1 Department of Psychology, School of Biological Sciences, University of Cambridge, Cambridge, CB2 1TN UK; 2 Yale Program on Climate Change Communication, Yale University, New Haven, CT 06520 USA

**Keywords:** Psychology, Environmental social sciences, Energy and society, Social sciences

**Arising from** C. F. Nisa et al. *Nature Communications*10.1038/s41467-019-12457-2 (2019)

The degree to which behavioral interventions can promote widespread action on climate change is an important and urgent question. In their recent meta-analysis, Nisa et al.^1^ conclude that behavioral interventions have alarmingly small effects. Here we re-analyze the data and conclude that the meta-analytic effect reported by Nisa et al.^1^ is highly sensitive to the chosen estimator and, across a range of alternative estimators, we find that the average effect-size of behavioral interventions is at least twice as large as initially reported. These results are important not only because they impact estimates of the potential for population-level behavior change but also because they have implications for how behavioral researchers should conduct meta-analyses.

We applaud Nisa et al.^1^ for their important meta-analysis on randomized controlled trials, which evaluated behavioral interventions to promote household action on climate change. We agree with the authors that no clear consensus exists on the question of which behavioral interventions are most effective in promoting individual and collective action on climate change. Yet, we were surprised and concerned to learn that across the 83 interventions published between 1976 and 2017 that met the inclusion criteria—which jointly included over 3 million individuals—the average standardized effect size across all behavioral interventions was only *d* = −0.093 (95% CI; −0.16, −0.06). This effect size is small with one side of the confidence interval relatively close to zero. The authors translate this effect into a probability of benefit (i.e., probability that the intervention will promote climate change mitigation behaviors in the experimental vs. control group) of 6.6%. The authors conclude: “behavioral interventions have a very small positive effect” and “our work indicates alarmingly low levels of behavioral plasticity” (p.7). Because this is a strong conclusion with policy-implications, it is important that the data support this claim.

Here we make the straightforward point that unfortunately the data do not support the conclusions reported by Nisa et al.^1^. In fact, the reader might be interested to learn that the results of the meta-analysis are highly sensitive to the estimation method. For example, the level of heterogeneity in the reported meta-analysis is very high (as measured by the *I*^2^ statistic), up to 98% for some interventions (e.g., transportation) with an overall average of about 65%. Nisa et al.^1^ estimated a random-effects meta-analysis with the DerSimonian–Laird (DL) procedure, a common method for modeling between-study variance. However, when heterogeneity is moderate to high—as is the case in the current study—this estimator is biased and not recommended^2^. Although the DL method is relatively simple and popular, it can lead to severe underestimation of the variance when either the number of studies is limited or the heterogeneity is large^3^. Instead, Empirical Bayes or Restricted Maximum Likelihood is often recommended^2^, especially when heterogeneity is relatively high.

When we re-estimate Nisa et al.'s^1^ meta-analysis using the data provided with Empirical Bayes we get a very different estimate for the average standardized effect size: *d* = −0.204 (95% CI; −0.250, −0.158). Importantly, the estimation method need not be limited to Empirical Bayes to make this point. For example, using other often recommended heterogeneity estimators^4^, such as restricted maximum likelihood (REML), we obtain a very similar estimate of *d* = −0.19 (95% CI; −0.24, −0.15). The Sidik–Jonkan procedure gives an estimate of *d* = −0.22 (95% CI; −0.27, −0.17) and Hedges yields a near-identical estimate of *d* = −0.21 (95% CI; −0.25, −0.16). Under the range of effect-sizes observed here, the probability of benefit (POB) more than doubles—from 6.6% to 14.4%.

This finding should therefore lead to different conclusions about the average efficacy of behavioral interventions. Indeed, based on these results, the conclusion of the paper should probably not be that behavioral interventions have very small effects or alarmingly low levels of behavioral plasticity. It is important to contextualize this difference, because effect sizes in psychology are known to be small^5^ and, as others have noted^6^, their effects can accumulate over time with repetition. Following Furukawa and Leucht^7^, we convert Cohen’s *d* into the number-needed-to-treat (NNT) using a default control group event rate of 20%. A *d* of 0.09 could be considered trivial and requires, on average, about 38 people to be exposed to the treatment to engender one extra unit of behavior change in the treatment group. Importantly, this number falls by more than 50% to only 16 when *d* = 0.20, which is generally considered a meaningful effect size in psychology^5^. In other words, we want to point out to the reader that the main results and overall conclusions of the paper are highly sensitive to the estimation method.

It would have been helpful if the authors had made a case for why their estimation method was appropriate in light of the significant degree of heterogeneity across interventions. Moreover, instead of relying on a single-estimator, the authors could have conducted robustness checks with multiple estimators to provide transparency for the reader around how the estimation method influences the results of the meta-analysis. Such sensitivity analyses are generally recommended as reliance on a single estimator in meta-analyses may lead to non-robust conclusions^4^. For example, as we show here, alternative estimators provide the reader with a more optimistic interpretation about the average efficacy and plasticity of behavioral interventions to promote household action on climate change. Although the average effects of behavioral interventions are still not particularly large in absolute terms (*d* = 0.20), they are not alarmingly small and could be consequential when scaled at population level^5^. At minimum, they are significantly larger than what was reported by Nisa et al.^1^.

## Data Availability

The data necessary to reproduce the analyses reported in this paper can be retrieved from: https://figshare.com/articles/Dataset_Nisa_et_al_2019_NComms_xlsx/9641999.
